# Compensatory Development and Costs of Plasticity: Larval Responses to Desiccated Conspecifics

**DOI:** 10.1371/journal.pone.0015602

**Published:** 2011-01-05

**Authors:** Asaf Sadeh, Noa Truskanov, Marc Mangel, Leon Blaustein

**Affiliations:** 1 Department of Evolutionary and Environmental Biology and the Institute of Evolution, Faculty of Natural Sciences, University of Haifa, Haifa, Israel; 2 Department of Biology, Faculty of Natural Sciences, University of Haifa, Haifa, Israel; 3 Department of Applied Mathematics and Statistics, Jack Baskin School of Engineering, University of California Santa Cruz, Santa Cruz, California, United States of America; 4 Department of Biology, University of Bergen, Bergen, Norway; University of Maribor, Slovenia

## Abstract

Understanding constraints on phenotypic plasticity is central to explaining its evolution and the evolution of phenotypes in general, yet there is an ongoing debate on the classification and relationships among types of constraints. Since plasticity is often a developmental process, studies that consider the ontogeny of traits and their developmental mechanisms are beneficial. We manipulated the timing and reliability of cues perceived by fire salamander larvae for the future desiccation of their ephemeral pools to determine whether flexibility in developmental rates is constrained to early ontogeny. We hypothesized that higher rates of development, and particularly compensation for contradictory cues, would incur greater endogenous costs. We found that larvae respond early in ontogeny to dried conspecifics as a cue for future desiccation, but can fully compensate for this response in case more reliable but contradictory cues are later perceived. Patterns of mortality suggested that endogenous costs may depend on instantaneous rates of development, and revealed asymmetrical costs of compensatory development between false positive and false negative early information. Based on the results, we suggest a simple model of costs of development that implies a tradeoff between production costs of plasticity and phenotype-environment mismatch costs, which may potentially underlie the phenomenon of ontogenetic windows constraining plasticity.

## Introduction

Phenotypic plasticity is a widespread phenomenon: individuals alter their phenotypes in response to environmental cues, often as an adaptation to variable environments. This multidisciplinary concept has recently been of increasing interest to biologists as a feature of both normal and abnormal individual development that is not only shaped by evolution, but also one that influences the function of individuals, the structure of ecological communities, and evolutionary trajectories [1], [2], [3], [4]. Despite its apparent adaptive superiority, phenotypic plasticity is neither universal nor infinite in expression. To arrive at a better understanding of its evolution, we must identify its costs and limits, and illuminate the functional relationships between them [4], [5], [6]. Auld et al [7] suggested that many of these limits and costs may be alternative views of the same constraint, arguing that most of them are merely special cases or consequences of two fundamental costs, phenotype-environment mismatch (costs of phenotypes) and costs of the ability to be plastic (primarily maintenance and production costs).

Phenotype-environment (P-E) mismatch results in an ecological cost and is often caused by imperfect cue reliability and/or developmental lag times in the induced traits. Some phenotypes (e.g. morphological as opposed to behavioral) require substantial lengths of time to be expressed after their induction has been triggered [8], and an early warning is necessary well before the anticipated condition occurs to avoid this cost. However, cue reliability tends to decline with the duration between the cue and the environmental condition it predicts. False early information can also induce the wrong phenotype for the eventual environmental condition [9]. Therefore, organisms are expected to rely on multiple cues [e.g. 10], that may be available at different times before the anticipated condition and with variable degrees of reliability. Such continuous integration of multiple cues from the environment may be limited by the problem of processing contradictory cues. An additional limitation that has been frequently observed or assumed in models is the restriction of developmental flexibility to certain ontogenetic windows, beyond which developmental trajectories become canalized [e.g. 11], [12]. However, general explanations for the occurrence of such windows are mostly lacking [but see 13].

In the absence of ontogenetic windows that limit the expression of plasticity, or within such windows, the contradiction of early information by late but more reliable information is expected to induce compensatory development, requiring the organism to express an extreme degree of plasticity. Accelerated development, while avoiding the cost of P-E mismatch, may incur greater costs of producing the target phenotype in the form of reduced life expectancy [14], compromised immune system [15], locomotor performance [16]. However, constraints on plasticity – both the onset of late ontogenetic canalization and the costs of compensatory development – may be asymmetrical for different developmental trajectories induced by false early information. In other words, compensating for a false alarm for a particular stress may be constrained to a different degree than compensating for the unexpected occurrence of the same stress.

Explaining patterns of size and age at life-history transitions such as metamorphosis has been an ongoing, central challenge in evolutionary biology. Larval development towards the completion of metamorphosis in amphibians involves two directional processes: growth in body size and the differentiation and remodeling of tissues and organs [17]. The rates of both of these processes generally respond to various environmental factors, ultimately determining size and age at metamorphosis, respectively, and have been the focus of extensive research involving analyses of phenotypic plasticity [e.g. 11], [12], [17], [18], [19], [20], [21], [22]. Some of this work has focused on developmental responses to the risk of habitat termination, with the overwhelming majority showing that larvae accelerate development and metamorphose earlier [reviewed in 23]. This response tends to result in a smaller size at metamorphosis due to a shortened growth period. However, few studies have explicitly addressed other, endogenous costs [e.g. 15], [24], particularly such that lead to increased mortality [25], [26].

We studied the responses of fire salamander larvae to two cues for habitat termination that differ in their timing and reliability, to test the following hypotheses: 1) larvae sense recent desiccation of conspecifics from previous cohorts as an early cue of their habitat's duration, and respond adaptively by altering their developmental rates to increase their probability of metamorphosing before it desiccates; 2) if developmental plasticity is not ontogenetically limited, rates of development will be updated according to later cues that indicate the habitat's actual duration more reliably, to the point of complete compensation; 3) in the latter case, environmental demand for extreme plasticity will carry asymmetric developmental costs. The acceleration of a biological process that requires energy inputs is intuitively expected to demand increased effort and thus incur greater costs. Therefore, we specifically predicted that compensatory, hyper-accelerated development following a false negative early cue (i.e. an unexpected catastrophe) will be more costly to execute than a compensatory delay in development following a false positive early cue (i.e. a false alarm). Based on the results of our experiment, we suggest a simple model of costs of development rates. We use it to demonstrate how phenotype production costs of plasticity are influenced by the timing of information and its reliability, and how production costs are traded off with P-E mismatch costs to explain the frequently-observed ontogenetic loss of plasticity.

## Methods

### Ethics statement

Field collection of salamanders, experimentation and their return were conducted according to the Nature and Parks Authority permit 2009/36605 and the Animal Experimentation Ethics Committee permit 190/10.

### Study organism

We studied the ovoviviparous fire salamander [*Salamandra infraimmaculata*; 27], whose larvae are deposited during the winter into mostly temporary pools in northern Israel. These pools vary greatly in various ecological characteristics, including their water holding capacity. Many temporary pools dry more than once within the same breeding season, particularly during early winter (October–December) and spring (March–April), when rains are infrequent, temperatures are high and the ground water level is low [28]. Pool desiccation is a very important factor contributing to salamander larval mortality and reproductive failure [29]. Many early-born larvae that die in early-winter events of pool desiccation dry in the sun (Sadeh, personal observations), with their flesh likely changing in chemical composition. Most of their decomposition occurs only after the pools are reflooded later in the season, possibly emitting unique chemicals that may be perceived by conspecifics. A pool's water holding ability depends on its floor structure, connectedness to the ground water table and exposure to solar radiation, and often does not change within a single breeding season. Thus, the presence or absence of such chemicals can convey moderately reliable information to the bulk of conspecifics that are deposited during mid-winter regarding the pool's liability to early-spring desiccation. Although previous experiments showed that *S. infraimmaculata* habitat selection behavior is finely tuned to changing ecological conditions [e.g. 30], a preliminary mesocosm experiment indicated that gravid females may not be responding to this cue in their choice of larviposition pool (Sadeh, unpublished data). Thus the larvae, deposited indiscriminatingly with regards to formerly desiccated larvae, are predicted to perceive this cue and respond to it by adjusting their development rates.

### Animal collection and return

To prepare the cue of recent desiccation, we collected larvae that either died in desiccated natural pools or in other mesocosm experiments during previous years (up to 3 years), dried them by placing under a light bulb until their mass stopped decreasing and stored them in sealed plastic bags at −20°C. Since no larva was intentionally killed to prepare this manipulation, the experiment was limited in size by the number of available dried larvae. In order to hasten their physical decomposition, we ground the dried larvae to a heterogeneously fine powder using a manual pestle and mortar after weighing 50 individuals to determine their mean individual dry mass. Thirty six hours before the experiment, we mixed powder quantities of 0.445 g (equivalent to 9 larvae) into outdoor tubs, each filled with 40 L tap water. The 36-hour waiting time allowed chlorine to dissipate from the water, and the powder to partially dissolve and initiate the organic decomposition process. *Salamandra* females often larviposit into pools within the first few days after they fill (Sadeh, personal observations). After this waiting time, we stirred the tubs and took water from them to fill the experiment's “early cue” treatment tubs. We took water for the “no early cue” treatment tubs from similar outdoor tubs that held 36-hour-aged tap water and did not contain any conspecific powder. With this manipulation, we did not control for the possible perception of the dried conspecifics by the focal larvae as a risk of predation, either by sensing their death (but not drying) as an alarm cue, or by sensing their presence (but not death) as older, cannibalistic conspecifics. However, the risk of predation is known in this species to induce reduced foraging and delayed development [31], [32; Sadeh, unpublished data], the opposite pattern than predicted for the perception of risk of desiccation.

During November 2009, we collected gravid females from natural breeding sites and placed them in field enclosures containing mesocosms [identical to those described in 30] to larviposit. We weighed and photographed each larva ventrally, and after removing larvae that were at the extremes of the mass distribution, we paired individuals from the same mother and of similar mass. Up to 10 hours after birth, we randomly allocated each pair to an experimental tub. Mean individual mass of the larvae at the beginning of the experiment was 0.243 g (SD = 0.041 g), and their mean snout-vent length (SVL) was 1.7 cm (SD = 0.1 cm). After giving birth, we returned all the adult females and excess larvae to their sites of collection. After the experiment ended, we returned all the surviving metamorphs to the natural pools nearest to the location of their mother's collection.

### Experimental design

**Figure 1:**
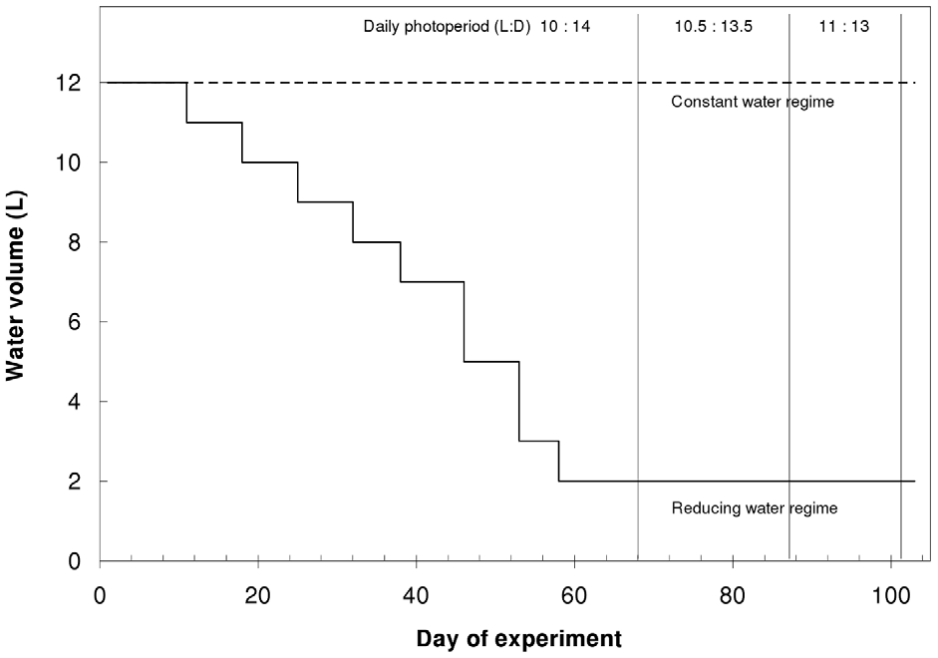
Experimental water volume regimes. Constant (dashed) and reducing (solid) water volumes over time. Vertical lines represent times of photoperiod increment by 30 minutes.

We conducted a factorial design experiment combining two levels of early cue of desiccation (presence/absence of dried conspecific powder, hereafter “early cue”) with two levels of water regime (constant/reducing water volume according to Figure 1). Thus, the experiment tested the developmental responses (growth and larval period) of larvae to the early information embodied in the presence or absence of the cue, both in situations when this early information was either true or false. We replicated each treatment combination 8 times in indoor, cuboid tubs (floor dimensions 36 by 21 cm), initially filled with 12 liters of water and containing 2 sibling larvae. We prepared the early cue as described above and implemented it as a single pulse manipulation before day 1. We removed water from the reducing water regime throughout the experiment by periodically filtering out water through coffee filter paper, and returning the residues from the filter paper back into the tub. This was also done to the constant water tubs but both residues and water were returned. We compensated weekly for water loss from constant water tubs due to evaporation by adding deionized water. The resulting increasing difference in solutes between treatments is a natural effect of pools drying partially due to evaporation. We only decreased the water volume to 2 liters in the reducing water regime because we sought to measure the larval response until metamorphosis without killing them or imposing a limitation on their swimming and feeding behavior. We fed the larvae *ad libitum* throughout the experiment to ensure similar food intake among treatments. This feeding regime also eliminated any potential chemical effects or indirect trophic effects of the introduced dried conspecifics material. We fed the larvae either a mixture of field-collected zooplankton organisms [mainly *Arctodiaptomus similis* (Copepoda), various daphnids and mosquito larvae (*Culiseta longiareolata* and *Culex laticinctus*)], or purchased chironomid (*Chironomus* sp) larvae.

We followed natural daily photoperiod in the experimental room, beginning with 10∶14 (L∶D) on day 1, 10.5∶13.5 from day 68, 11∶13 from day 87, and 11.5∶12.5 from day 101 to the end of the experiment at day 103. Similarly, we kept temperatures at the region's long-term mean daily maximal temperature for each month, according to the Israel Meteorological Service website (http://www.ims.gov.il/IMSEng/CLIMATE). Temperatures were: 16–18 degrees during days 1–76, 17–19 degrees during days 77–99 and 19–21 degrees from day 100. However, on days 76 and 85, due to air conditioning system malfunction, air temperatures rose to 36°C and 26°C, respectively, for 1–2 days before the problem was corrected, equally affecting the water temperatures of all treatments. The first case of air conditioning failure marked the beginning of emergence of metamorphs for this experiment. While these sharp deviations in temperature were not planned, they occurred during springtime when severe heat waves occur naturally in Israel, raising air and water temperatures to yearly extrema of up to 40°C and affecting natural ponds in a similar way when not completely drying them.

### Response variables and metamorphosis

We recorded larval mortality and growth prior to metamorphosis, as well as size at and time to metamorphosis. To determine growth, we weighed all the larvae to the nearest mg and photographed them on days 1, 5 (weight only), 22, 42, 58, 76, 81, 87, 95, 101 and 103. Once the first larvae started displaying progressive metamorphic morphology (dark skin color, a reduced tailfin and/or reduced gills), we checked the tubs every one to two days to collect emerging metamorphs. We photographed emerging metamorphs to determine size at metamorphosis, and recorded their times to metamorphosis. The photographs were used to determine SVL to the nearest mm using image processing software (ImageJ 1.40g). Body mass is a good index of an individual's immediate condition and short-term growth as it includes the mass gained by recent meals that may be stored in lipid reserves or quickly used up. Therefore it is quick to respond to environmental conditions and carries relatively high intra-individual variation (Sadeh, personal observations). In contrast, SVL is slower to respond and less sensitive to short-term conditions, but gives a better estimate of long-term growth, as it is the result only of the portion of energy that was allocated into skeletal development and growth in body size.

### Statistical analyses

We used repeated measures ANOVA to test the effects of the early cue and water regime on larval growth trajectories during most of their growth period, both in mass (five dates) and in SVL (four dates), using tub means as independent data points. The repeated measures analysis was done up to day 58, before any larva metamorphosed to prevent the distortion of test results by the reductions in mean sizes due to the removal of the usually larger emerged larvae. However, considerable shifts in the response patterns occurred during the metamorphic period. The results of these shifts are captured in a two-way ANOVA used to test the effects of early cue and water regime on time to- and size (SVL) at metamorphosis. We removed from this analysis two tubs in which both larvae died. To test our hypotheses regarding the costs of the expression of plasticity, we used three orthogonal planned contrasts on mean larval mortality data [33]: the main effect of water reduction (the mean of the two reducing water treatments vs. the mean of the two constant water treatments), the effect of a false positive early cue (early cue + reducing water vs. no early cue + reducing water) and the effect of a false negative cue (no early cue + constant water vs. early cue + constant water).

## Results

**Figure 2:**
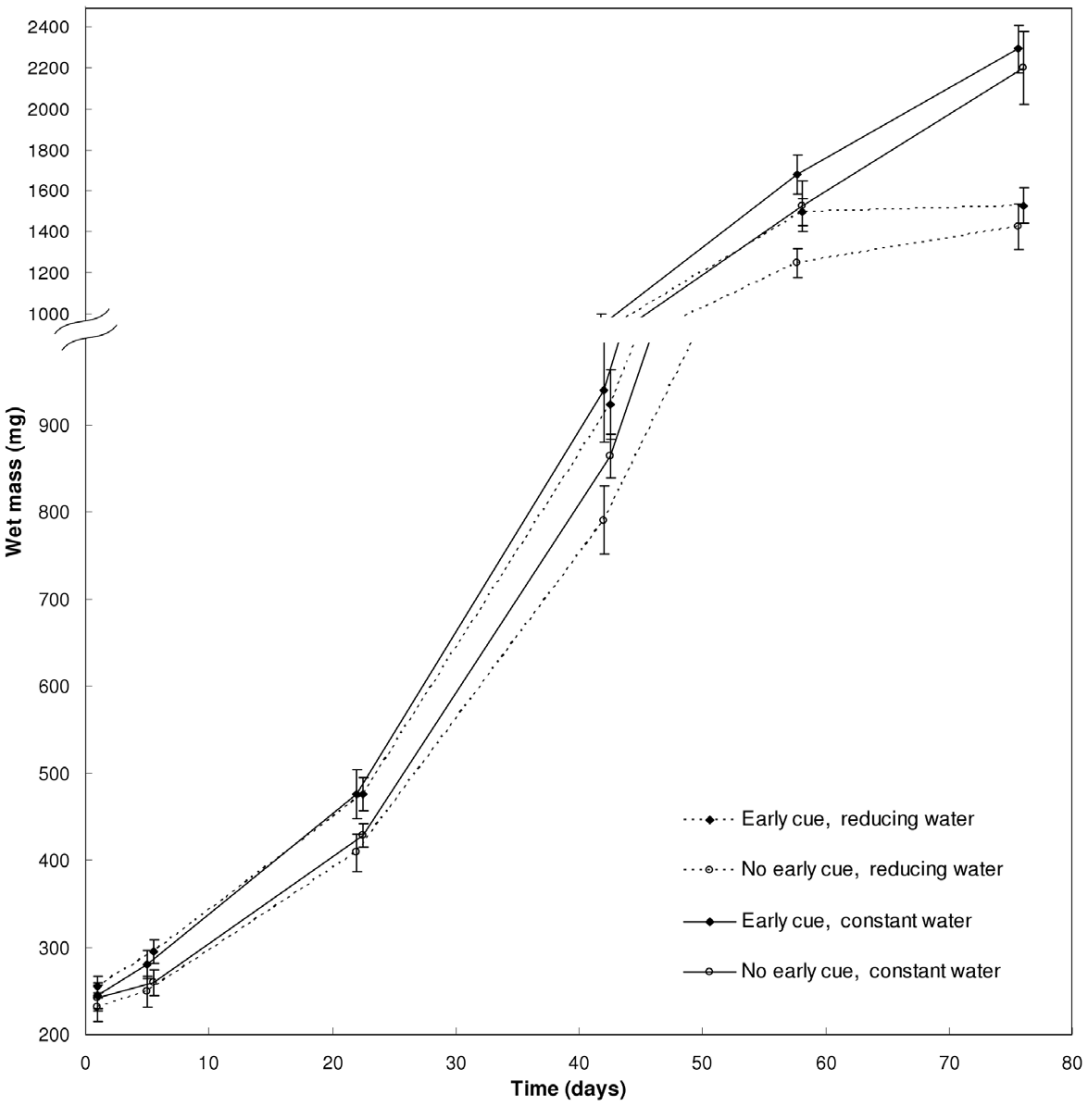
Mean larval growth trajectories in wet mass. The ordinate axis was rescaled at 1000 mg to magnify the patterns during the early larval period, where the early cue demonstrated an accelerating effect on development. Error bars are SE.

The overall mass growth during the first 58 days (prior to any metamorphosis) was positively affected by the early cue (p = 0.016). There was no significant effect yet of the water regime, during this period, nor a significant early cue × water regime interaction (Table 1; Figure 2). The mass growth trajectory was positively affected by both water volume regime (time × water regime interaction: p = 0.001) and the early cue (time × early cue interaction: p = 0.008), but not by their interaction (time × water regime × early cue interaction: p = 0.876; Table 1). Qualitatively similar results were obtained for the test of these factors' effects on larval SVL growth (Table 2).

**Table 1:** Results for a repeated measures ANOVA test on larval mass growth up to day 58.

Between subjects	SS	df	MS	F	P
Early Cue	222494.514	1	222494.514	6.520	0.016
Water regime	99326.139	1	99326.139	2.911	0.099
Early cue × Water regime	31823.702	1	31823.702	0.933	0.342
Error	955471.619	28	34123.986		

Within subjects	Wilks' λ	Hypoth. df	Error df	F	P
time	0.016	4	25	396.654	<0.0005
time × early cue	0.586	4	25	4.406	0.008
time × water regime	0.465	4	25	7.205	0.001
time × early cue × water regime	0.954	4	25	0.298	0.876

**Table 2:** Results for a repeated measures ANOVA test on larval SVL growth up to day 58.

Between subjects	SS	df	MS	F	P
Early Cue	0.238	1	0.238	7.147	0.012
Water regime	0.061	1	0.061	1.836	0.186
Early cue × Water regime	0.044	1	0.044	1.315	0.261
Error	0.932	28	0.033		

Within subjects	Wilks' λ	Hypoth. df	Error df	F	P
time	0.006	3	26	1498.046	<0.0005
time × early cue	0.543	3	26	7.300	0.001
time × water regime	0.555	3	26	6.953	0.001
time × early cue × water regime	0.991	3	26	0.076	0.973

The larvae responded quickly to the early cue of desiccation risk by accelerating their mass growth rates (Figure 2). An *a posteriori* t-test of larval mass on day 5 of the experiment revealed that the early cue has already produced a significant effect (t = 2.219, p = 0.034). By day 22, larvae in the treatment combinations that received a positive early cue grew to a larger mean size compared to those of treatments without this cue (a 13% difference). Growth rates considerably slowed under the reducing water level regime after day 42, and came to almost a complete cessation following day 58 (Figure 2) due to the cessation of feeding during metamorphic climax. Under the constant water level regime this growth restriction was evident only after day 76. Thus, the water level regime gained an increasing effect on growth that overwhelmed the effect of the early cue only near metamorphosis, after day 58 (Figure 2).

**Figure 3:**
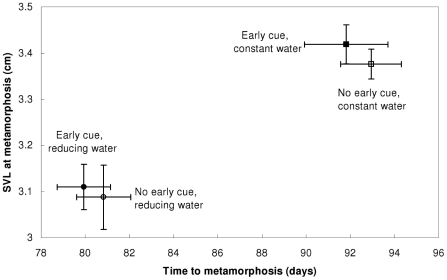
Final phenotype reaction norms of mean time to- and size at metamorphosis. Only the water level regime exerted a lasting effect on final phenotypes. Error bars are SE.

**Figure 4:**
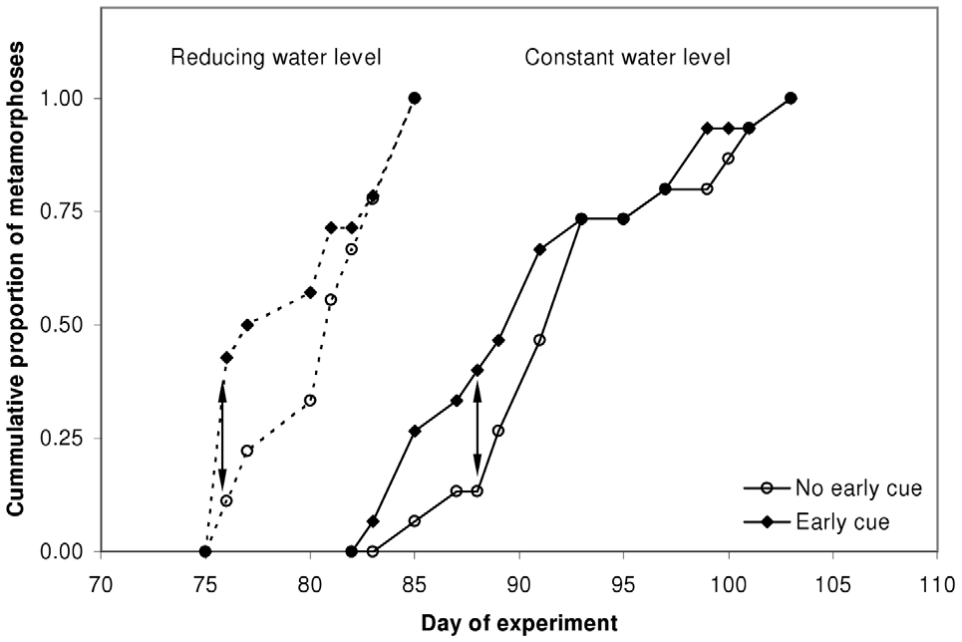
Cumulative proportions of individual metamorphoses over time. Metamorphoses in the ‘early cue’ treatments were skewed to the right compared to the ‘no early cue’ treatments in both water level regimes. Arrows indicate the times of maximal distances between the cumulative distributions.

Time to emergence responded significantly only to the water volume regime (Figure 3; two-way ANOVA: P<0.0005; Table 3), with a LS mean emergence time of 80.4 days for the reducing water volume treatments and 92.4 days for the constant water volume treatments. The early cue effect and the water regime × early cue interaction term were both non-significant, indicating that the larvae compensated for their initial response to early information according to prevailing hydroperiod conditions. Though the central tendencies of the early cue treatments did not differ significantly from those without the cue, their mean times to metamorphosis were slightly shorter under both water regimes. The temporal distribution of individual emergences revealed an initial surge of metamorphoses in both of the treatment combinations with the early cue compared to treatments without it (Figure 4). In both water level regimes, the maximal difference between cumulative metamorph frequencies of the two early cue treatments occurred at the first quartile (day 76 and day 88 in the reducing water and the constant water regimes, respectively). *A posteriori* comparisons between the proportions of metamorphs at these times between early cue treatments showed that this initial surge was significant in both water level regimes (reducing water regime: Z = −1.750, p = 0.040; constant water regime: Z = −1.703, p = 0.044; one-sided normal approximation tests of equality of proportions). Thus, the initial developmental response to the early cue of desiccation risk may have skewed the distribution of emergences to the left, but was mostly compensated for when eventually contradicted by prevailing conditions.

**Table 3:** Results for two-way ANOVA tests on SVL at- and time to metamorphosis.

SVL at metamorphosis	Sum-of-Squares	df	Mean-Square	F-ratio	P
Early cue	0.008	1	0.008	0.458	0.504
Water regime	0.659	1	0.659	38.887	0.000
Early cue × water regime	0.001	1	0.001	0.046	0.832
Error	0.440	26	0.017		

Time to metamorphosis	Sum-of-Squares	df	Mean-Square	F-ratio	P
Early cue	7.539	1	7.539	0.451	0.508
Water regime	1061.539	1	1061.539	63.559	0.000
Early cue × water regime	0.097	1	0.097	0.006	0.940
Error	434.240	26	16.702		

Similar to the overall pattern of time to metamorphosis, final larval sizes at metamorphosis only differed significantly between water level regimes (P<0.0005; Figure 3; Table 3), with a LS mean SVL of 3.1 cm in the reducing water volume treatments and 3.4 cm in the constant water volume treatments. This pattern indicates that increased allocation of energy to hastened differentiation and/or a shorter growth period compromised the total larval growths, whereas initial responses in development rates to false early information on risk of desiccation were compensated for by later responses to actual hydroperiod conditions.

**Figure 5:**
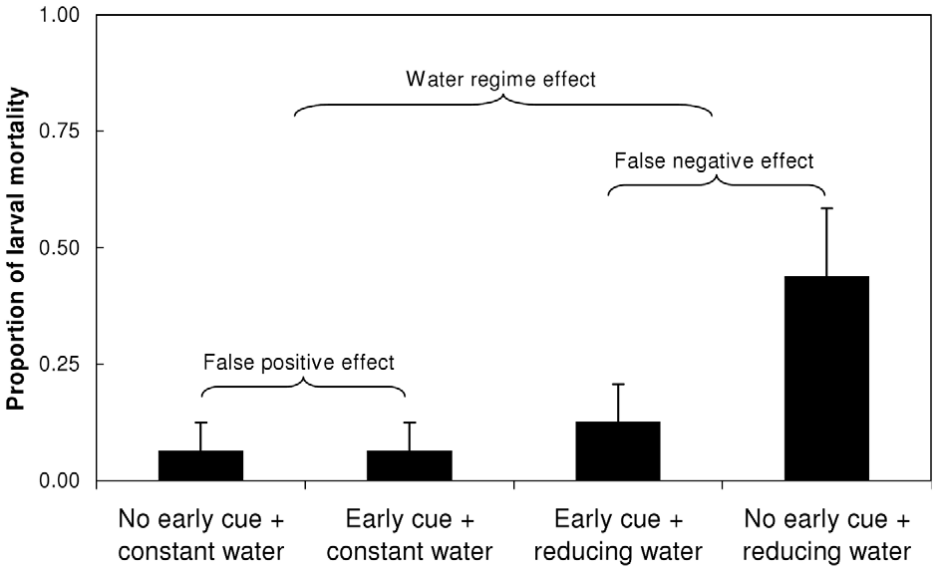
Larval mortality prior to metamorphosis. Mortality rates were greater in the reducing, compared to the constant water level regime. Within this regime, the false negative early cue caused considerably greater mortality. No effect was found for the false positive early cue. All larval mortality occurred after day 75 of the experiment.

Larval mortality was most pronounced under the reducing water regime without an early cue (43.75%). Under the reducing water regime and with the early cue mortality rate was 12.5%, whereas in the other treatment combinations, mortality rates were 6.25% (Figure 5). The reducing water regime significantly increased mortality compared to the constant water regime, regardless of the early cue (P = 0.029). However, when the water loss was unexpected, mortality increased significantly (by 31.25%; P = 0.028) compared to the same condition when preceded by the presence of the early cue. In contrast, mortality under the constant water regime was similar following a false early cue or its absence (6.25%; P = 1.000). See Table 4 for the statistical summary of these contrasts. No larval mortality occurred before day 75.

**Table 4:** Results for planned contrasts on larval mortality.

Contrast	Sum-of-Squares	df	Mean-Square	F-ratio	P
Water regime	0.383	1	0.383	5.277	0.029
False negative early cue	0.391	1	0.391	5.385	0.028
False positive early cue	0.000	1	0.000	0.000	1.000
Error	2.031	28	0.073		

## Discussion

### Responses to and compensation for early cues

The results support the hypothesis that the recent desiccation of conspecifics serves as an early cue for risk of pool desiccation, accelerating development at least via early larval growth (Figure 2). Since it is generally agreed that size thresholds limit differentiation rates [20], [21], [23], an early cue indicating a potentially severe time constraint on development and requiring its acceleration is predicted to induce accelerated growth so that differentiation remains unconstrained. This response is opposite to that of larvae under risk of predation or cannibalism [31; Sadeh, unpublished data], and therefore we rule out the larvae's perception of dried-up conspecifics as a cue for predation/cannibalism risks.

No staging system has been developed for this species to indicate its ontogenetic progress. However, gradual morphological changes (in skull shape, skin color and limb usage patterns) occur throughout larval ontogeny before the conspicuous final stage of metamorphosis (Sadeh, personal observations), indicating that differentiation is occurring throughout the larval period, along with growth in body size [consistent with 17], [19], [34]. Therefore, similar to growth rate, differentiation rate is likely also hastened by the early cue for desiccation. Our *a posteriori* analysis of the temporal distributions of metamorphoses (Figure 4) suggests that the early cue had an accelerating effect on the rates of differentiation under both water regimes, but this effect did not last to significantly affect mean time to metamorphosis as a result of full developmental compensation by most of the individuals for the false early information.

By the end of the larval period, the effects of the early cue had practically vanished, with only the water regime exerting a strong effect. This is not surprising since the dynamics of water depth, water volume and concentration of solutes are far more reliable cues for future desiccation than the scent of recent death by desiccation. Larvae markedly compensated for their initial response to false early information according to prevailing hydroperiod conditions, showing considerable developmental plasticity that is not limited to early phases of the larval period. This was evident by practically identical reaction norms for both size at- and time to metamorphosis for true and false early cues, under both water regimes (Figure 3).

### Costs of developmental rates

Compensatory development was mostly apparent between days 42 and 76 (Figure 2), followed by increased mortality (Figure 5), that occurred only after day 76 and to the end of the experiment. The water reduction regime induced a high average rate of development (larval period^−1^), regardless of the presence of the early cue, associated with increased mortality compared to the constant water regime. However, a significantly greater contribution to this increased mortality was due to the compensation demanded by the unexpected reduction in water, in the absence of the early cue, where the larvae had to hyper-accelerate their development. In contrast, we found no detectable costs of reducing the developmental rate to compensate for an early false alarm. This pattern of mortality suggests that it was the result of the maximum instantaneous rate of development performed by the larvae.

Recent studies have found that high growth rates and especially compensatory growth are traded-off with other life-history traits and body functions over various time scales [35], including lifespan [14], reproductive output [36] and locomotor performance [37]. This possibly occurs through the accumulation of cellular damage caused by oxidative stress, or through increased allocation of resources to its repair [38], [39], [40] at the expense of other functions. For example, a study by Inness & Metcalfe [14] suggested that three-spined sticklebacks that reproduce only once in their lifetime, cannot afford to divert resources away from reproduction in order to repair damage inflicted by compensatory growth. Therefore, fish under these conditions suffered increased rates of mortality. A similar tradeoff may underlie the pattern of mortality in our experiment; metamorphosis requires high inputs of energy [41] and cannot be delayed or compromised to repair the damage caused by compensatory development when the larval habitat approaches termination. In contrast to compensation in growth rates, little work has been done on compensatory differentiation rates, and their costs are less understood. Accelerated differentiation of stem cells may reduce the available pool of undifferentiated cells and limit other functions they may serve (Artyom Kopp, personal communication). At the tissue level, Arendt & Hoang [42] suggested that accelerated differentiation of muscle tissue results in numerous but smaller fibers and reduced performance of the tissue. At the whole-organism level, some tradeoffs for accelerated differentiation rates have been identified in amphibians, with the effects sometimes carried beyond metamorphosis [26], such as decreased immune function [15] and locomotor performance [16]. These costs may stem from adaptive allocation of limited resources to various body functions, or from compromised whole-organism coordination of different tissues, resulting in disruption of homeostasis and increased vulnerability to environmental stress. Studies on heat shock protein expression also indicate that stress resistance and development appear to be negatively related [reviewed in 39]. Indeed, mortality in our experiment may have resulted also from a compromised ability to cope with the accidental heat waves that occurred in our lab, similar to those that frequently occur in nature late in the larval period.

### Costs and limits of plastic phenotype development

Callahan et al [6] emphasized the need to distinguish costs of phenotypes from costs of plasticity *per se*, and to address their potential interactions. We agree with this important distinction, but see no reason why the fundamental production cost of phenotypes should differ between plastic and fixed development. Development is a cumulative process. Therefore, the total phenotype production cost for a certain trait value is the sum of costs incurred during each small time interval throughout its development. We suggest that this cost may *accumulate* differently throughout the development of a trait to incur increased costs for plastic development. Based on our results and the growing body of recent literature on the costs of accelerated, compensatory development, we will show that the instantaneous production cost can be generally characterized as an increasing, strictly convex function of the instantaneous rate of development.

Consider an organism that must develop a certain trait, *p*, from an initial state of *p* = 0 to some required final state, *p_req_*, within a limited time interval, 0≤*t*≤*T*, where *T* is unknown and must be assessed. Furthermore, in order to minimize ecological phenotype-environment mismatch costs, the organism must reach *p_req_* at time *T* exactly. For example, an aquatic larva that is born into an ephemeral pond must progress the differentiation of body systems for terrestrial function while growing, and complete metamorphosis before the pond dries to avoid death by desiccation. However, upon completion of metamorphosis it must emerge from the water and miss further opportunities for larval growth in case this occurs too early. Thus, the organism must continuously assess its time limit and adjust its rate of development, *r*, accordingly, such that
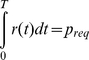
(1)The following analysis can equivalently correspond to cases where the time for development is fixed with the organism having to assess the expected environmental conditions at that time and match the target trait value accordingly (e.g. produce defenses against an uncertain predation level). Either way, the problem is that of adjusting developmental rates. However, development incurs costs. Defining the development rate-dependent instantaneous production cost, 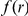, the cumulative cost of producing the required trait is
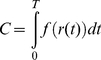
(2)The results of our experiment, as well as recent literature on compensatory development imply that
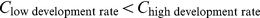
(3a)

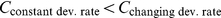
(3b)Equation (3a) clearly implies that 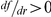 for any value of *r*. We will now show that Eqn. (3b) implies that 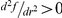 for any value of *r*.

At *t* = 0, the organism perceives mildly reliable information on its future time limitation, and sets an initial rate of development, *r*_1_, accordingly (or this can be a genetically-determined default rate in the absence of any information). Unless additional information is perceived later by the organism, allowing it to reassess the remaining time available for development, this rate will remain constant. Assume that at time *t_c_* (0<*t_c_*<*T*) the organism perceives a perfectly reliable information on the time limitation, allowing it to readjust its developmental rate to *r*_2_, such that Eqn. (1) is satisfied. Thus,
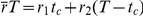


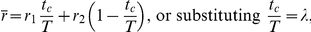


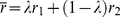
(4)is the required average rate of development over the entire time interval [0 *T*] to attain *p_req_*, where 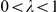 is the relative weight of the duration of development at the rate of *r*_1_. In reality, organisms continuously perceive and integrate multiple cues that bear imperfect information and readjust their developmental rates, resulting in more curved developmental trajectories than assumed in our analysis. However, these curved trajectories can be approximated as a sequence of many short linear intervals similar to those considered here.

From Eqn. (3b) we know that
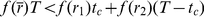
(5)Substituting (4) into (5) we get:
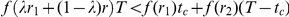


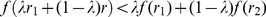
(6)Equation (6) is the mathematical definition of strict convexity for 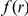. In other words, 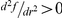 for any value of *r*.

That 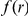 is a strictly increasing and convex function has important implications for life-history tradeoffs of development. Specifically, as reliable information regarding the required developmental rate of a trait is perceived later in that trait's ontogeny, complete compensation demands increasing endogenous costs, to the point that they may exceed the ecological costs of phenotype-environment mismatch that compensation is aimed at minimizing. This would not have been the case if 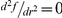, since then (3b) would be an equality, and the cost for any given final phenotype would be constant regardless of the developmental trajectory leading to it. Thus, for any plastic trait our analysis predicts that at some point in its ontogeny, development will become canalized and cease to respond to environmental cues that otherwise induce its acceleration. The specific timing of loss of plasticity depends on the specific forms of 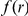 and of the phenotype-environment mismatch cost as a function of the deviation of the realized phenotype from the required phenotype, and can only be considered in a full, system-specific life-history model. Ontogenetic loss of plasticity has been documented frequently in various organisms [e.g. 11], [12], [13], [43], and we suggest that our analysis provides a potential general explanation for this phenomenon. In contrast, compensatory deceleration of development demands little costs. In this case, the extra costs are incurred for the needlessly high initial rates of development before the perception of corrective information. Therefore, the deceleration of development is not predicted to be limited by endogenous costs throughout ontogeny.

**Figure 6:**
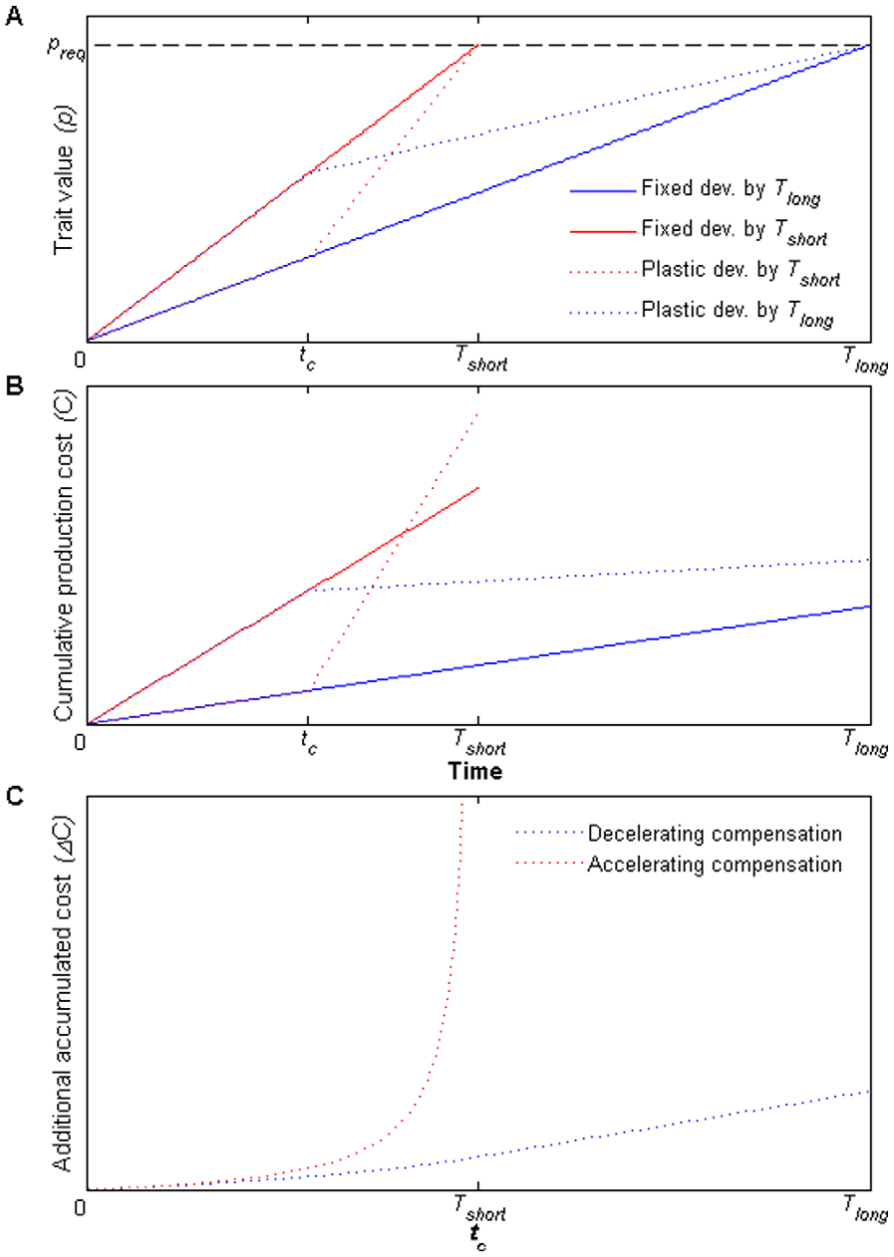
Trait developmental trajectories and their production costs. (A) A simulation of trait development trajectories towards a required trait value, *p_req_*, under time limitations. Red and blue lines complete development by *T_short_* and *T_long_*, respectively. Solid lines stand for fixed developers that do not or cannot change their developmental rate. Dotted lines stand for plastic developers that change their developmental rate upon perception of information at *t_c_*. (B) Cumulative production costs for the corresponding developmental trajectories. (C) The additional accumulated costs of compensatory development as a function of the time of perception of reliable information. Red and blue lines accelerate or decelerate their initial rates of development to attain the required trait value at *T_short_* or *T_long_*, respectively.

To graphically illustrate the model's behavior and implications, we arbitrarily chose a function that upholds the general requirements of 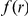, i.e. an increasing and convex function of *r*:
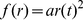
(7)where *a* is a scaling coefficient. In Figure 6A we simulate the developmental trajectories of a fixed slow developer, a fixed fast developer, as well as plastic developers that accelerate or decelerate their developmental rates following the reception of perfectly reliable cues at some point. Figure 6B demonstrates how production costs accumulate for these developmental trajectories, calculated according to equations (2) and (7), resulting in greater costs for fast developers, and increased costs for the expression of compensatory development. Figure 6C shows the increase in the extra production costs incurred by compensatory development (the production cost of plasticity), as reliable information is perceived later. While the extra production cost for accelerating compensation approaches infinity as reliable information is perceived later towards the time limit, it only approaches a finite value in the case of decelerating compensation.

Our model predicts a lower cost of compensating for a “false alarm” than for an “unexpected catastrophe”. However, in our experiment, we found no evidence for any cost for the former. Detecting endogenous costs empirically is very difficult, since they could manifest in different body functions, through adaptive tradeoffs or physiological outcomes. Mortality is the ultimate cost, but it bears information only on the extreme cases of physiological compromise, below which costs may remain undetected. Thus, sub-lethal costs may have been incurred in our experiment, but remained below our detection threshold.

We suggest that phenotype production costs accumulate to greater costs for individuals expressing plastic development since they do not follow the most efficient trajectory towards their final phenotype. Therefore, early information is extremely valuable for reducing deviations from the most efficient trajectory, but it tends to be less reliable. Complex and more effective information acquisition strategies [e.g. plants extrapolating temporal dynamics into the future; 44] are expected to evolve to moderate this tradeoff, but these strategies and the maintenance of their underlying physiological mechanisms may themselves be costly [5].

Analysis of constraints on phenotypic plasticity has mostly utilized final-phenotype reaction norms, comparing trait values at the end of their development over different environments, thus capturing the phenotype-environment relationship at the end-point of the underlying developmental process. Clearly, more may be learned about plasticity and its constraints by considering the development of the ultimate phenotype and the role of plasticity in it [13], [45], [46]. Our study motivates future studies to manipulate the timing of perceived cues and their reliability throughout the ontogeny of the focal phenotype, as well as to determine the system-specific proximate mechanisms of costs of developmental rates at the cellular, tissue and whole-organism levels. Such combined ecological and developmental approaches, with system-specific life-history modeling, hold a promise for advancing our understanding of developmental plasticity, its costs and evolution.
